# Complete Permittivity Tensor in Sputtered CuFe_2_O_4_ Thin Films at Photon Energies between 2 and 5 eV

**DOI:** 10.3390/ma6094096

**Published:** 2013-09-16

**Authors:** Martin Veis, Roman Antos, Stefan Visnovsky, Prasanna D. Kulkarni, Narayanan Venkataramani, Shiva Prasad, Jan Mistrik, Ramanathan Krishnan

**Affiliations:** 1 Faculty of Mathematics and Physics, Charles University in Prague, Prague 12116, Czech Republic; E-Mails: antos@karlov.mff.cuni.cz (R.A.); visnov@karlov.mff.cuni.cz (S.V.); 2 Department of Physics, Indian Institute of Technology, Bombay 400076, India; E-Mails: prasanna@tifr.res.in (P.D.K.); shiva.pd@gmail.com(S.P.); 3 Department of Metallurgical Engineering & Material Science, Indian Institute of Technology, Bombay 400076, India; E-Mail: ramani@iitb.ac.in (N.V.); 4 Faculty of Chemical Technology, Univerzity of Pardubice, Pardubice 53210, Czech Republic; E-Mail: jan.mistrik@upce.cz (J.M.); 5 Groupe d’Etude de la Matiére Condensée, CNRS, Université Versailles St. Quentin, F-78035 Versailles, France; E-Mail: lalikri@aol.com (R.K.)

**Keywords:** spectroscopic ellipsometry, magneto-optics, spinel ferrite, CuFe_2_O_4_, permittivity tensor

## Abstract

This work is devoted to the systematic study of the optical and magneto-optical properties of sputter deposited CuFe${}_{2}$O${}_{4}$ thin films in the photon energy region between 2 and 5 eV using spectroscopic ellipsometry and magneto-optical Kerr spectroscopy. The spectral dependence of both the diagonal and off-diagonal elements of the permittivity tensor is determined. A complete picture about the electron transitions in CuFe${}_{2}$O${}_{4}$ is suggested in the frame of intervalence charge transfer and intersublattice charge transfer transitions. The effect of deposition conditions and post-deposition treatment in CuFe${}_{2}$O${}_{4}$ films upon the optical and magneto-optical properties is discussed.

## 1. Introduction

Recent conceptions of integrated photonic devices, which allow a high-speed data transmission, require the integration of elements that have non-reciprocal effects, such as optical isolators [1] or circulators [2]. Current materials widely used to fabricate non-reciprocal bulk components are ferrimagnetic garnets, which are non-compatible with current silicon technology. This significantly complicates the on-chip integration. Very recently, a novel design of magneto-optical waveguide-based cobalt ferrite nanoparticles embedded in silica matrix has been proposed [3]. Such a kind of magneto-optical photonic device has great promise to be easily integrated on a silicon chip. However, a detailed knowledge of optical and magneto-optical properties of ferrite material is crucial to properly design the device structure with an improved figure of merit.

Spinel ferrites with the general formula, MFe${}_{2}$O${}_{4}$ (M = Mn, Cu, Zn, Ni, Co, Mg, *etc*.), are chemically stable, widely studied materials, due to their potential applications in biomedicine, solar cells, magneto-optical displays, high density magneto-optical recording, electro-magnetic wave absorbers, color imaging, gas sensing, *etc*. [4,5,6,7]. Copper ferrite, CuFe${}_{2}$O${}_{4}$, formed by the substitution of Fe${}^{2+}$ ions in isostructural Fe${}_{3}$O${}_{4}$ by Cu${}^{2+}$ ions, is one of the candidates for possible applications in novel non-reciprocal devices. The cation distribution can be described as (Cu${}_{\alpha }^{2+}$Fe${}_{1-\alpha }^{3+}$)[Cu${}_{1-\alpha }^{2+}$Fe${}_{1+\alpha }^{3+}$]O${}_{4}^{-2}$, where parentheses and square brackets denote tetrahedral and octahedral sites, respectively. The ground state of an octahedrally coordinated Cu${}^{2+}$ ion is ${}^{2}E_{g}{\left(t_{2g}\right)}^{6}{\left(e_{g}\right)}^{3}$, while the only excited state is ${}^{2}T_{2g}{\left(t_{2g}\right)}^{5}{\left(e_{g}\right)}^{4}$ [8]. The inversion of the level order leads to the important effect on the ${}^{2}$E${}_{g}$ level, which is highly susceptible to a Jahn-Teller configurational instability [9] that removes the degeneracy of the ground state. Therefore, the octahedral Cu${}^{2+}$ ions tend to suffer from a tetragonal distortion, and the tetragonal phase copper ferrite (c/a∼1.06), which is completely inverse, is the only one stable at room temperature. A phase transition from the tetragonal to the cubic lattice occurs at temperatures of about 390 ${}^{\circ }$C [10]. A special post-deposition treatment, which consists of an annealing procedure at high temperatures and subsequent quenching in liquid nitrogen, therefore, allows a preparation of cubic phase CuFe${}_{2}$O${}_{4}$ thin films [11].

There have been several attempts to describe the optical and magneto-optical properties of various spinel ferrites in terms of absorption bands. Owing to the complex electronic structure, a confusing variety of interpretations has been reported, because of different assignments of observed transitions. Recent studies adopted the picture of intervalence charge transfer (IVCT) and intersublattice charge transfer (ISCT) transitions proposed by Scott [12]. In both IVCT and ISCT transitions, an electron is transferred from one cation to a neighboring cation on the same or a different crystallographic site. Unlike the crystal field transitions, the IVCT transition involves two cations, which results in the relaxed parity selection rule [12]. Therefore, a higher oscillator strength is expected for IVCT transition contrary to the crystal field and orbital promotion transitions.

Previous magneto-optical studies of CuFe${}_{2}$O${}_{4}$ have been done employing the Faraday effect in the photon energy range between 0.5 and 2.5 eV [13] and the Kerr effect in the photon energy range between 2 and 5 eV [14,15]. Kim *et al.* [16] assigned the spectroscopic structures observed in the polar Kerr spectra of CuFe${}_{2}$O${}_{4}$ to particular electron transitions. Unlike the permittivity tensor, the Kerr spectra are not directly related to the electronic structure, and such assignment is only approximative. To the best of the authors knowledge, there is no systematic study on the spectral dependence of the complete permittivity tensor of CuFe${}_{2}$O${}_{4}$. However, information about the spectral dependence of the complete permittivity tensor provides important information about the electronic structure of the material and is necessary for the design of novel photonic devices based on CuFe${}_{2}$O${}_{4}$.

In this paper, we present a systematic attempt to investigate the electronic structure of copper ferrites using the experimental techniques of spectroscopic ellipsometry and magneto-optical spectroscopy. Coherently with the results presented on various types of ferrite compounds, we carefully describe all optical transitions revealed by experiment. Moreover, we discuss the influence of the structural change of CuFe${}_{2}$O${}_{4}$ on magneto-optical properties.

## 2. Experimental Section

CuFe${}_{2}$O${}_{4}$ thin films were RF sputtered on 10×10 mm polished fused quartz substrates using a tetragonal copper ferrite target, which was prepared by the conventional ceramic technique. The base pressure in a vacuum chamber was $6\times 10^{-7}$ mbar. The film deposition was carried out in Ar + O${}_{2}$ gas mixture at working pressure $6\times 10^{-3}$ mbar, and the oxygen to argon ratio was maintained at 15%. The RF power was 50 and 200 W at 13.6 MHz. The substrates were neither heated nor cooled during the sputtering. After the deposition, the samples were annealed at 850 ${}^{\circ }$C for two hours and, then, slowly furnace-cooled (SC samples) or quenched (Q samples) in liquid nitrogen.

The crystallographic structure and magnetic properties of deposited films were studied by a Philips PW 1729 X-ray diffractometer (XRD) and a vibrating sample magnetometer (VSM) [10]. XRD studies revealed peaks typical for cubic spinel structure (c/a = 1), in the case of the quenched sample, and peaks typical for tetragonal structure (c/a∼1.05), in the case of slowly cooled samples. This is consistent with the knowledge that the copper ferrite can be transformed from the low temperature tetragonal to the high temperature cubic phase only at temperatures higher than 390 ${}^{\circ }$C [17]. VSM measurements showed the highest saturation magnetization, $M_{S}$, in the quenched sample and the lowest in the as deposited film [10]. The quenched sample exhibits 35% higher $M_{S}$ than the bulk value ($M_{S\;\text{bulk}}$ = 1700 G [18]), indicating the Cu${}^{2+}$ cation redistribution. The parameters of investigated samples are summarized in Table 1.

**Table 1:** Basic parameters of the set of CuFe${}_{2}$O${}_{4}$ samples. $T_{A}$ denotes the annealing temperature and $M_{S}$ denotes the saturation magnetization.

Sample	Thickness [nm]	RF power [W]	$4\pi M_{S}$ [G]	$T_{A}$ [${}^{{}^{\circ}}C$]	Structure
1—Quenched	112	50	2300	850	Cubic
2—As deposited	90	50	750	–	As deposited
3—Slowly cooled	280	50	1500	850	Tetragonal
4—Slowly cooled	230	200	1600	850	Tetragonal

The magneto-optical spectroscopy was carried out using an azimuth modulation technique with synchronic detection in polar and longitudinal configuration. The experiment has been done in the photon energy range between 1.2 and 4.6 eV. The experimental optical set up included a 450 W high power Xe arc lamp, quartz prism monochromator, polarizer, DC compensating Faraday rotator, AC modulating Faraday rotator, phase plate (for Kerr ellipticity measurements), sample in magnetic field, analyzer and photomultiplier [19]. In the small angle approximation, the complex polar magneto-optical Kerr effect was measured at nearly normal light incidence, as a ratio of $\theta_{K}+i\varepsilon_{K}\approx \left(r_{yx}/r_{xx}\right)$ of Jones reflection matrix elements, where $\theta_{K}$ and $\varepsilon_{K}$ are the Kerr rotation and ellipticity. The longitudinal magneto-optical Kerr effect was measured similarly at the angle of incidence, adjusted to 72 degrees for p-polarized incident light as a ratio of $\theta_{K}+i\varepsilon_{K}\approx \left(r_{ps}/r_{pp}\right)$ [20]. The applied magnetic field was 470 mT and 100 mT in the polar and longitudinal configuration, respectively. In both configurations, the magnetic field was sufficient for the film saturation (as was checked by the measurement of the magnetic field dependence of the magneto-optical Kerr effect). During the polar configuration measurements, the samples were placed on a water-cooled pole piece of electromagnet, and their temperature was stabilized at 285 K. In the longitudinal configuration measurements, the samples were kept at the stabilized room temperature of 295 K. The effect of stray magnetic field on the optics was accounted for using an Al reflector.

Theoretical models of the magneto-optical Kerr effect have been calculated employing transfer matrix formalism [21]. In polar magnetization and normal light incidence, a layer of CuFe${}_{2}$O${}_{4}$ was characterized by the permittivity tensor:

(1) $$\varepsilon \approx \left[\begin{array}{ccc} \varepsilon_{1} & j\varepsilon_{2} & 0\\ -j\varepsilon_{2} & \varepsilon_{1} & 0\\ 0 & 0 & \varepsilon_{1} \end{array}\right]$$

where all elements have real and imaginary part: $\varepsilon_{j}=\varepsilon_{j}^{\prime }-i\varepsilon_{j}^{\prime \prime }$. The diagonal element, $\varepsilon_{1}$, is related to the normal refractive index, *n*, and the normal extinction coefficient, *k*. The off-diagonal element is related to the refractive index and the extinction coefficient of right and left polarized light.

A four-zone null ellipsometer was employed to obtain the spectral dependences of ellipsometric parameters in the spectral range from 1.5 to 5.4 eV. To increase the accuracy of measured data processing, the spectra were recorded for three angles of incident light at 65${}^{\circ }$, 70${}^{\circ }$ and 75${}^{\circ }$. Because the fused quartz substrate was side polished, incoherent back-reflections from the backside of the substrate contributed to the measured signal and complicated the optical characterization. Therefore, a liquid solution procedure (LSP) [22], in which a small amount of wadding paper infused by a mixture of glycerin and water, optically matched to the quartz, was attached to the bottom of the substrate to avoid the back-reflections.

## 3. Results and Discussion

### 3.1. Spectroscopic Ellipsometry

**Figure 1:**
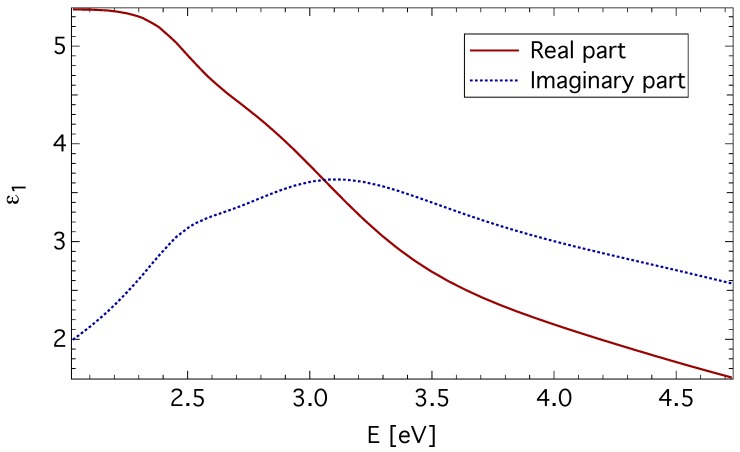
The diagonal element, $\varepsilon_{1}$, of the permittivity tensor of quenched CuFe${}_{2}$O${}_{4}$ thin film (Sample 1).

The spectral dependence of $\varepsilon_{1}$ was parametrized by the sum of four damped Lorentz oscillators, and the least square method was employed to adjust the film thickness, the transition energy, strength and broadening for each oscillator. The spectral dependence of $\varepsilon_{1}$ obtained for the quenched CuFe${}_{2}$O${}_{4}$ sample is displayed in Figure 1. It is similar in shape to dependences reported on Fe${}_{3}$O${}_{4}$ [23], CoFe${}_{2}$O${}_{4}$ [24] and MgFe${}_{2}$O${}_{4}$ [25], indicating a similar electronic structure.

Spectroscopic ellipsometry revealed four optically active transitions centered around 2.4, 3.1, 4.8 and 13.2 eV. The first three transitions were also observed by magneto-optical experiments. The differences in energies are small and are within the experimental data errors. Therefore, we postpone the discussion of these transitions to the next section and focus here only on the transition centered near 13.2 eV.

The separation energy of about 5–10 eV between the valence band of oxygen 2p orbitals and the 4s orbital of the transition metal ions has been reported in various transition metal oxides [26,27]. Strong absorption above 8 eV has been reported by Zhang *et al.*, [25] in optical reflection measurements on Mg and Li ferrites. Alvarado *et al.* [28] reported the same spectral behavior in photoelectron-spin-polarization measurements, with photon energies up to 11 eV on Fe${}_{3}$O${}_{4}$. This suggests that the electric-dipole allowed transition between the O${}^{2-}$ 2p valence band and the Fe${}^{3+}$ 4s conduction band is responsible for the spectral structure near 13.2 eV.

The obtained spectra of $\varepsilon_{1}$ were subsequently used in the calculations of the off-diagonal element of the permittivity tensor, $\varepsilon_{2}$, from the magneto-optical Kerr measurements.

### 3.2. Magneto-Optical Spectroscopy

#### 3.2.1. Polar Geometry

**Figure 2:**
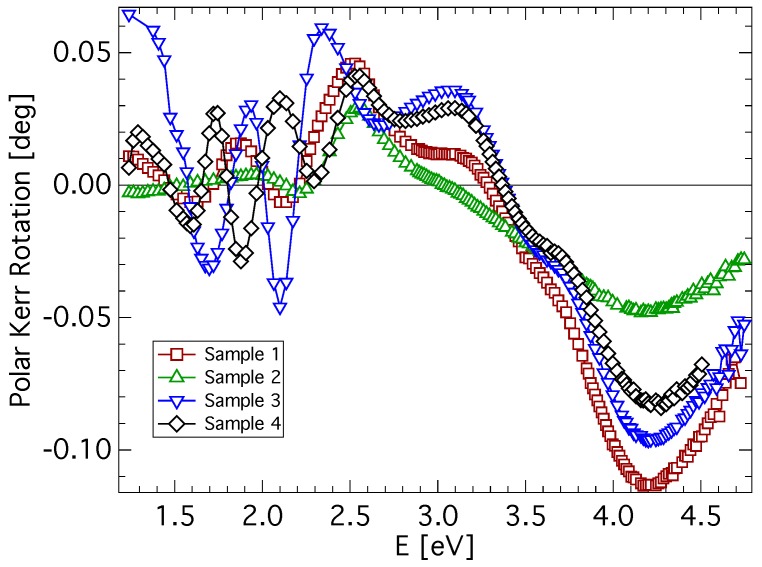
Polar Kerr rotation spectra of CuFe${}_{2}$O${}_{4}$ thin films measured at nearly normal incidence.

**Figure 3:**
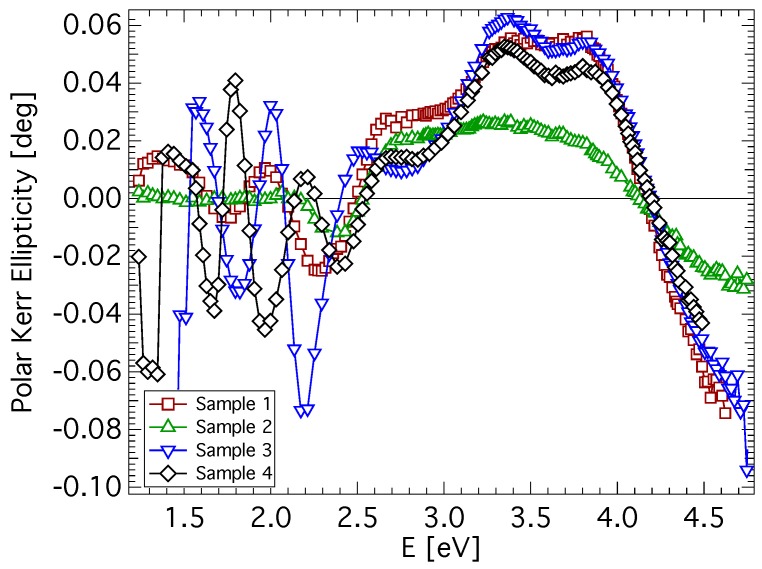
Polar Kerr ellipticity spectra of CuFe${}_{2}$O${}_{4}$ thin films measured at nearly normal incidence.

Experimental spectra of polar Kerr rotation, $\theta_{K}$, and ellipticity, $\varepsilon_{K}$, of all investigated samples are shown in Figure 2 and Figure 3. A low level of noise in the spectra reflects the very good quality of CuFe${}_{2}$O${}_{4}$ films. All samples exhibit similar spectral behavior of the polar Kerr effect with only minor differences. A contribution of the propagation across the film resulting in the interference is clearly visible in the photon energy range below 2.4 eV. Besides, the polar Kerr rotation spectra are dominated by two visible peaks with opposite signs near 3.1 and 4.2 eV. On the other hand, polar Kerr ellipticity spectra show positive peaks near 3.5 and 3.8 eV. The amplitudes of the polar Kerr effect differ with the sample, which is due to the different $M_{S}$ (see Table 1). The highest amplitude is exhibited by the quenched sample, while the lowest amplitude is exhibited by the as-deposited sample. The increase in $\theta_{K}$ when the sample is quenched is the consequence of its transformation from the tetragonal to the cubic structure. In cubic copper ferrite, migration of cupric ions to the tetrahedral site causes an increase in the magnetization [11,29]. Reduced magnetic moment and the smallest MO amplitude of the as-deposited sample points to the presence of a nanocrystalline form of CuFe${}_{2}$O${}_{4}$ [30,31]. Smaller grain size and large grain boundary volume leads to the suppression of exchange interactions responsible for the spin ordering in the lattice. Polar Kerr spectra show spectral behavior similar to that reported by Kim *et al.*, [16] on samples prepared by the sol-gel method (note the different convention in the definition of magneto-optical parameters).

**Figure 4:**
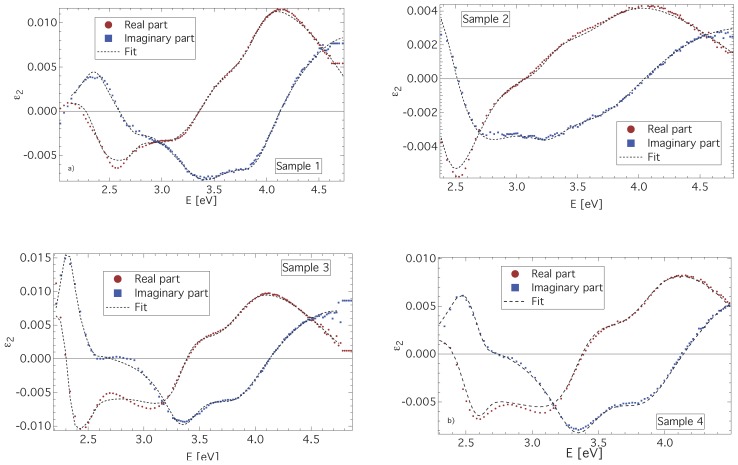
The off-diagonal elements, $\varepsilon_{2}$, of CuFe${}_{2}$O${}_{4}$ thin films deduced from the polar Kerr measurements along with the fitted theoretical dependence.

To get a deeper insight into the magneto-optical properties of CuFe${}_{2}$O${}_{4}$ thin films, a spectral dependence of $\varepsilon_{2}$ has been deduced from the polar Kerr measurements, considering a model structure of a thin CuFe${}_{2}$O${}_{4}$ layer on a semi-infinite quartz substrate. The spectral dependence of $\varepsilon_{2}$ for all investigated samples in the photon energy range from 2 to 4.8 eV is displayed in Figure 4. All samples exhibit similar spectral behavior of $\varepsilon_{2}$, with only minor differences. The most departing spectrum appears to be that of the as-deposited sample (Sample 2). This is, however, acceptable with respect to the similar differences in XRD and magnetic measurements. Nevertheless, all spectra of the real part of $\varepsilon_{2}$ exhibit negative peaks near 2.6 and 3.1 eV and a broad positive peak near 4.2 eV. On the other hand, spectra of the imaginary part of $\varepsilon_{2}$ are dominated by two positive peaks near 2.5 and 4.7 eV and a negative spectroscopic structure composed of two peaks near 3.3 and 3.9 eV. Such spectral dependences are similar to those reported on MgFe${}_{2}$O${}_{4}$ bulk samples [32], as well as to those reported on Li${}_{0.5}$Fe${}_{2.5}$O${}_{4}$ single crystals [25,33,34]. Martens *et al.* [24] reported experimental results on Co ferrite, but those results differ from the results presented in this paper.

The spectral dependences of $\varepsilon_{2}$ derived from the magneto-optical measurements were parametrized by a summation of five paramagnetic line shapes [35], and the least square method was employed to adjust the energy, strength and broadening for each transition. The resulting fits are included in Figure 4, and the fitting parameters are summarized in Table 2.

**Table 2:** The magneto-optically active transitions in CuFe${}_{2}$O${}_{4}$ thin films between 2.0 and 5.0 eV. Listed are the transition energy, $\omega_{0}$, linewidth, $\Gamma_{0}$, intensity, ($\varepsilon_{2}^{\prime \prime }$)${}_{max}$, and transition assignment. (Note that the parentheses denote the tetrahedral coordination and the square brackets the octahedral coordination.) ISCT, intersublattice charge transfer; IVCT, intervalence charge transfer.

Sample	1	2	3	4
($\varepsilon_{2}^{\prime \prime }$)${}_{max}$	0.0053	0.0072	0.0163	0.0075
$\omega_{0}$ [eV]	2.36	2.35	2.31	2.44
Γ [eV]	0.19	0.21	0.10	0.18
Transition I	ISCT $\left(Fe^{3+}\right)t_{2}\to \left[Fe^{3+}\right]t_{2g}$			
($\varepsilon_{2}^{\prime \prime }$)${}_{max}$	−0.0017	−0.0052	−0.0012	−0.0026
$\omega_{0}$ [eV]	2.73	2.66	2.55	2.64
Γ [eV]	0.14	0.44	0.07	0.11
Transition II	ISCT $\left[Fe^{3+}\right]e_{g}\to \left(Fe^{3+}\right)e$			
($\varepsilon_{2}^{\prime \prime }$)${}_{max}$	−0.0087	−0.0019	−0.0092	−0.0079
$\omega_{0}$ [eV]	3.34	3.25	3.34	3.33
Γ [eV]	0.43	0.27	0.22	0.25
Transition III	ISCT $\left[Fe^{3+}\right]e_{g}\to \left(Fe^{3+}\right)t_{2}↑$			
($\varepsilon_{2}^{\prime \prime }$)${}_{max}$	−0.0063	−0.0029	−0.0081	−0.0061
$\omega_{0}$ [eV]	3.89	3.72	3.84	3.86
Γ [eV]	0.27	0.49	0.35	0.33
Transition IV	ISCT $\left(Fe^{3+}\right)t_{2}\to \left[Fe^{3+}\right]e_{g}↓$			
($\varepsilon_{2}^{\prime \prime }$)${}_{max}$	0.0099	0.0038	0.0087	0.0073
$\omega_{0}$ [eV]	4.74	4.71	4.66	4.50
Γ [eV]	0.72	0.98	0.72	0.53
Transition V	IVCT $\left[Cu^{2+}\right]e_{g}\to \left[Fe^{3+}\right]t_{2g}$			

A good agreement between the theoretical fit and the data deduced from the experiment is clearly visible from Figure 4. The fitting revealed five paramagnetic lines centered near 2.4, 2.7, 3.3, 3.9 and 4.6 eV.

A comparison of the $\varepsilon_{2}$ spectra with the results reported on MgFe${}_{2}$O${}_{4}$ [32], NiFe${}_{2}$O${}_{4}$ [36] or Li${}_{0.5}$Fe${}_{2.4}$O${}_{4}$ [25,33,34] helped to estimate which transitions are related to the iron ions and which are related to the copper ions.

The spectroscopic structure near 2.4 eV has been similarly observed in other ferrite compounds [25,32] and Fe${}_{3}$O${}_{4}$ [37], which indicates that such transition should involve only Fe${}^{3+}$ electrons [23]. Therefore, it is assigned to the ISCT transition, $\left(Fe^{3+}\right)t_{2}\to \left[Fe^{3+}\right]t_{2g}$, between tetrahedral and octahedral Fe${}^{3+}$ ions.

The paramagnetic line shape centered near 2.7 eV is not clearly visible in the $\varepsilon_{2}$ spectra, due to smaller oscillator strength. The most pronounced is this structure in sample 2. All spinel ferrites exhibit such a kind of spectroscopic structure in the vicinity of 2.6 eV. Clearly visible is the peak in the $\varepsilon_{2}$ spectrum of Li${}_{0.5}$Fe${}_{2.4}$O${}_{4}$ reported by Zhang *et al.* [25]. Since there is no change in energy with different ion substitution for this transition, only Fe${}^{3+}$ ions should be involved. Fontijn *et al.* predicted an ISCT transition in Fe${}_{3}$O${}_{4}$ near 2.64 eV [23]. Therefore, this structure is assigned to the ISCT transition, $\left[Fe^{3+}\right]e_{g}\to \left(Fe^{3+}\right)e$, between octahedral and tetrahedral Fe${}^{3+}$ ions.

The spectroscopic structures in the $\varepsilon_{2}$ spectra centered near 3.3 and 3.9 eV were observed across all ferrite compounds [23,25,37], including CuFe${}_{2}$O${}_{4}$ [16]. Consistently with previous reports, these structures were assigned to ISCT transitions between octahedral and tetrahedral sites, $\left[Fe^{3+}\right]e_{g}\to \left(Fe^{3+}\right)t_{2}↑$ and $\left(Fe^{3+}\right)t_{2}\to \left[Fe^{3+}\right]e_{g}↓$, respectively. The 3.3 eV structure is attributed to an ISCT transition in the majority spin bands, while the 3.9 eV one, to an ISCT transition in the minority spin bands.

These two transitions are responsible for the magneto-optical properties of CuFe${}_{2}$O${}_{4}$ in the spectral range between 2.8 and 3.5 eV. In this region, the slowly cooled (tetragonal) samples exhibit higher Kerr amplitudes than the quenched (cubic) sample. This is related to considerably higher amplitude ($\varepsilon_{2}^{\prime \prime }$)${}_{max}$ and broadening of transition IV in these samples. Since this transition involves tetrahedral $\left(Fe^{3+}\right)t_{2}$ ions, the migration of Cu${}^{2+}$ ions to tetrahedral sites in the cubic sample causes the decrease of $\left(Fe^{3+}\right)t_{2}$ ions per unit volume, resulting in the smaller oscillator strength and, consequently, the Kerr effect amplitudes.

Unlike in the previous cases, the energy of the spectroscopic structure centered near 4.4∼4.7 eV noticeable varies with the sample. This is a consequence of the decreased accuracy of the fit procedure, due to the end of the measured spectral region. Moreover, in the UV region, the magneto-optical Kerr measurement suffers from a higher level of noise, due to the increased role of light scattering.

There is no comparable transition reported in Li${}_{0.5}$Fe${}_{2.4}$O${}_{4}$ [25], NiFe${}_{2}$O${}_{4}$ [36] and CoFe${}_{2}$O${}_{4}$ [24], which points to the contribution of Cu${}^{2+}$ ions. IVCT transitions between divalent substituted ion and trivalent iron ion, both situated at octahedral sites, were observed in Co and Ni ferrites, $\left[Co^{2+}\right]t_{2g}\to \left[Fe^{3+}\right]t_{2g}$, at about 2.2 eV, and $\left[Ni^{2+}\right]t_{2g}\to \left[Fe^{3+}\right]t_{2g}$, at about 3.1 eV. Owing to the larger binding energies of more localized 3d electrons of Cu${}^{2+}$ compared to Co${}^{2+}$ and Ni${}^{2+}$, a similar transition is expected at higher energies. Considering the inversion of the electron level order for Cu${}^{2+}$ ions, the last spectroscopic structure was assigned to an IVCT $\left[Cu^{2+}\right]e_{g}\to \left[Fe^{3+}\right]t_{2g}$ transition.

In the presented results, we did not observe crystal-field transitions of Cu${}^{2+}$ ions, which are expected to be around 2.5 eV [38]. Such transitions are spin-allowed, but owing to the inversion symmetry at octahedral sites (which have a strong preference for Cu${}^{2+}$ ions in CuFe${}_{2}$O${}_{4}$), they are parity forbidden, resulting in their small oscillator strength. Therefore, these transitions are not visible in the presented spectra.

As follows from Table 2, the slowly cooled sample sputtered at 50 W RF power exhibits a considerably higher amplitude ($\varepsilon_{2}^{\prime \prime }$)${}_{max}$ of transitions (except Transition II) than the sample sputtered at 200 W RF power. This might indicate a decomposition of the target material at higher sputtering powers, which results in the decrease of exchange interactions and a lower number of active absorbing centers per unit volume. This is expected to cause the decrease of the transition strength.

On the other hand, the transitions in the quenched sample are broader compared to those in slowly cooled samples. It seems that this has a connection with the migration of cupric ions to the tetrahedral site. Because the center of the symmetry is missing at the tetrahedral sites, electron orbitals are more opened, and covalent bonding is increased, which results in the broadening of the transition line shapes. However, more detailed structural and optical studies are necessary to confirm this hypothesis.

Finally, we make a comment on the crystal field (CF) splitting energy, $\Delta_{CF}$, of tetrahedral and octahedral Fe${}^{3+}$ ions in CuFe${}_{2}$O${}_{4}$ thin films. As follows from Table 2, the crystal field energy splitting for the octahedral Fe${}^{3+}$ iron, $\Delta_{CF}^{O}$, is about 1.5 eV, while in the case of tetrahedral Fe${}^{3+}$ iron, $\Delta_{CF}^{T}$, it is about 0.7 eV. These values have been obtained as differences in transition energies. Camphausen *et al.* [26] reported that the octahedrally coordinated Fe${}^{3+}$ ions give $\Delta_{CF}^{O}=1.7-2.0$ eV, while the tetrahedrally coordinated Fe${}^{3+}$ ions give $\Delta_{CF}^{T}=0.86-1.17$ eV. Kim *et al.* [37] reported the value of $\Delta_{CF}^{O}=1.4$ eV. A reasonable agreement between presented $\Delta_{CF}$ values and previously published studies has been found. This confirmed the correctness of the assignment of the spectroscopic structures observed in $\varepsilon_{2}$ spectra to the particular transitions.

### 3.3. Longitudinal Geometry

**Figure 5:**
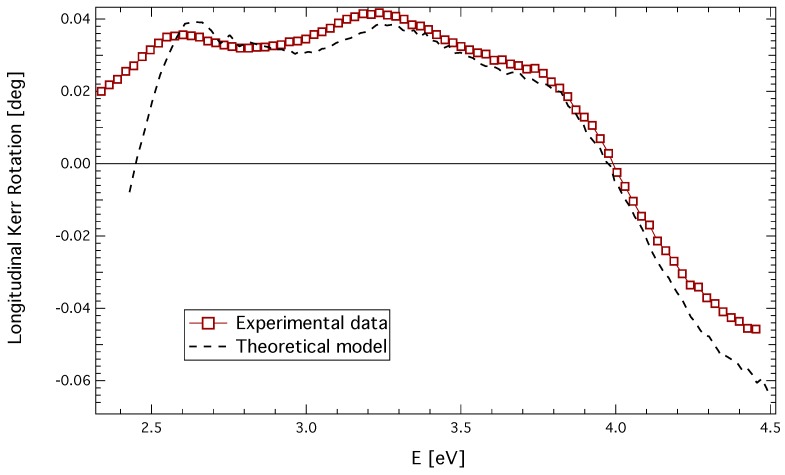
Experimental and theoretical longitudinal Kerr rotation of quenched CuFe${}_{2}$O${}_{4}$ thin film.

A longitudinal Kerr rotation spectrum of the quenched sample is shown in Figure 5. The spectrum exhibits three spectroscopic structures centered near 2.6, 3.3 and 3.9 eV. Theoretical calculation of the longitudinal Kerr rotation was performed utilizing the complete knowledge of the permittivity tensor of the CuFe${}_{2}$O${}_{4}$ layer. The resulting spectrum is also shown in Figure 5. A very good agreement between the experimental values and the theoretical calculation is clearly visible. Small differences in the IR and UV region are due to the thickness inhomogeneity, as well as the surface roughness of the sample.

## 4. Conclusions

Optical and magneto-optical properties of sputtered CuFe${}_{2}$O${}_{4}$ thin films with cubic and tetragonal structures have been investigated. The spectral dependence of the complete permittivity tensor has been derived in the photon energy range between 2 and 5 eV, and the influence of the post-deposition treatment on the magneto-optical properties of studied films was discussed. The combination of spectroscopic ellipsometry and magneto-optical spectroscopy revealed six spectroscopic structures in a broad spectral region near energies of 2.4, 2.7, 3.3, 3.9, 4.6 and 13.2 eV. The first five structures were described in the frame of ISCT and IVCT transitions between Fe${}^{3+}$ and Cu${}^{2+}$ ions. The last structure was discussed as an electron transfer between the O 2p valence band and the Fe 4s conduction band. Such assignment was confirmed by the derivation of the crystal field splitting energy for both octahedral and tetrahedral iron ions, respectively. The obtained energies reasonably agree with theoretically predicted values, as well as with experimental results obtained on similar compounds.
